# PARP inhibition and pharmacological ascorbate demonstrate synergy in castration‐resistant prostate cancer

**DOI:** 10.1002/1878-0261.70183

**Published:** 2026-01-14

**Authors:** Nicolas Gordon, Peter T. Gallagher, Orly I. Richter, Neermala Poudel Neupane, Amy C. Mandigo, Jennifer J. McCann, Emanuela Dylgjeri, Irina Vasilevskaya, Christopher McNair, Channing J. Paller, Wm. Kevin Kelly, Karen E. Knudsen, Matthew J. Schiewer, Ayesha A. Shafi

**Affiliations:** ^1^ Department of Cancer Biology Jefferson University Philadelphia PA USA; ^2^ Center for Prostate Disease Research, Murtha Cancer Center Research Program, Department of Surgery Uniformed Services University of the Health Sciences Bethesda MD USA; ^3^ The Henry M. Jackson Foundation for the Advancement of Military Medicine, Inc. Bethesda MD USA; ^4^ Department of Oncology Johns Hopkins University Baltimore MD USA; ^5^ Department of Medical Oncology Thomas Jefferson University Philadelphia PA USA; ^6^ Sidney Kimmel Cancer Center Thomas Jefferson University Philadelphia PA USA; ^7^ The American Cancer Society Philadelphia PA USA; ^8^ Department of Urology Thomas Jefferson University Philadelphia PA USA; ^9^ Department of Pharmacology/Physiology/Cancer Biology Thomas Jefferson University Philadelphia PA USA

**Keywords:** ascorbate, drug combinations, novel therapeutics, PARP, prostate cancer

## Abstract

Prostate cancer (PCa) is the second leading cause of cancer‐related death among men in the United States. While organ‐confined disease has a reasonable expectation of cure, metastatic PCa is universally fatal upon recurrence during hormone therapy, a stage termed castration‐resistant prostate cancer (CRPC). Until such time as molecularly defined subtypes can be identified and targeted using precision medicine, it is necessary to investigate new therapies that may apply to the entire CRPC population. The use of ascorbate, more commonly known as ascorbic acid or Vitamin C, has demonstrated antitumor activity in a variety of cancer cell types. There are several mechanisms currently under investigation to explain how ascorbate exerts anticancer effects. A simplified model depicts ascorbate as a pro‐drug for reactive oxygen species (ROS), which accumulate intracellularly and generate DNA damage. It was therefore hypothesized that poly (ADP‐ribose) polymerase (PARP) inhibitors, by inhibiting DNA damage repair, would augment the toxicity of ascorbate, leading to improved antitumor effects. Two distinct CRPC models were found to be sensitive to physiologically relevant doses of ascorbate. Moreover, additional studies indicate that ascorbate inhibits CRPC growth *in vitro* via multiple mechanisms including disruption of cellular energy dynamics and accumulation of DNA damage. Combination studies were performed in CRPC models with ascorbate in conjunction with escalating doses of three different PARP inhibitors (niraparib, olaparib, and talazoparib). The addition of ascorbate augmented the toxicity of all three PARP inhibitors and proved synergistic effects with olaparib in both CRPC models. Finally, the combination of olaparib and ascorbate was tested *in vivo* in both castrated and noncastrated models. In both cohorts, the combination treatment significantly delayed tumor growth compared to monotherapy or untreated control. These data indicate that pharmacological ascorbate is an effective monotherapy at physiological concentrations and kills CRPC cells. Ascorbate‐induced tumor cell death was associated with disruption of cellular energy dynamics and accumulation of DNA damage. The addition of PARP inhibition increased the extent of DNA damage and proved effective at slowing CRPC growth both *in vitro* and *in vivo*. These findings implicate ascorbate and PARPi as a novel therapeutic regimen that has the potential to improve CRPC patient outcomes.

## Graphical abstract

Pharmacologic ascorbate (vitamin C) increases ROS, disrupts cellular metabolism, and induces DNA damage in CRPC cells. These effects sensitize tumors to PARP inhibition, producing synergistic growth suppression with olaparib *in vitro* and significantly delayed tumor progression *in vivo*. Pyruvate rescue confirms ROS‐dependent activity. Together, these data identify ascorbate and PARP inhibition as a potential therapeutic strategy for advanced prostate cancer.

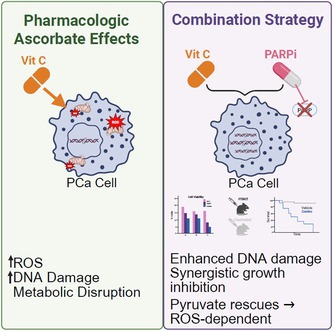

**Abbreviations**
ADT: androgen deprivation therapy
AR: androgen receptor
ATCC: American Type Culture Collection
ATP: adenosine triphosphate
BER: base excision repair
CPDR: Center for Prostate Disease Research
CRPC: castration‐ resistant prostate cancer
DDR: DNA damage repair
DMEM: Dulbecco's Mmodified Eagle Mmedium
DoD: Department of Defense
DSB: double‐strand break
dsDNA: double‐strand DNA
FDA: Food and Drug Administration
FFPE: formalin‐fixed, paraffin‐embedded
H&E: hematoxylin and eosin
HJF: Henry Jackson Foundation for the Advancement of Military Medicine, Inc
HR: homologous recombination
HRD: homologous recombination deficiency
HRR: homologous recombination repair
IACUC: Institutional Animal Care and Use Committee
IF: Immunofluorescence
IHC: Immunohistochemistry
IMEM: Improved Minimum Essential Medium
IP: intraperitoneal
mCRPC: metastatic castration‐resistant prostate cancer
NADPH: nicotinamide adenine dinucleotide phosphate
PARP: poly (ADP‐ribose) polymerase
PARPi: PARP inhibitors
PBS: phosphate‐buffered saline
PCa: prostate cancer
PFS: progression‐free survival
ROS: reactive oxygen species
SEM: standard error of the mean
SKCC: Sidney Kimmel Cancer Center
SSB: single‐strand break
TET: ten‐eleven translocation
TJU: Thomas Jefferson University
USUHS: Uniformed Services University of the Health Sciences

## 1 Introduction

The American Cancer Society projects 288,300 new cases of prostate cancer (PCa) will be diagnosed and estimates that PCa will result in 34,700 deaths in 2023 [1]. Most prostate cancer‐related deaths occur due to metastatic dissemination. Metastatic PCa responds to androgen deprivation therapy (ADT), sometimes referred to as chemical or medical castration; however, the cancer often recurs within 2–3 years [2]. At this point, the disease is termed metastatic castration‐resistant prostate cancer (mCRPC). Treatment options for patients with mCRPC are limited to more recently developed anti‐androgen agents such as enzalutamide and abiraterone, as well as radium‐223, docetaxel, and cabazitaxel; however, these agents are typically not curative [3, 4]. In short, the mCRPC population is in dire need of new therapeutic options.

The use of ascorbate, more commonly known as Vitamin C, in cancer treatment has been controversial for decades. In the 1970s, Linus Pauling and Ewan Cameron demonstrated a survival benefit for patients treated with ascorbate in a variety of end‐stage cancers [5]. Subsequent studies failed to show any benefit; however, and the concept of ascorbate as an anticancer agent was abandoned [6]. More recent research has revealed significantly limited bioavailability for orally administered ascorbate which explains why Cameron and Pauling's results with intravenous ascorbate were not replicated in the consequent studies designed to assess the benefits of oral supplementation. Intravenous administration of ascorbate can safely result in plasma levels as high as 20 mm, far beyond what is necessary to kill cancer cells [7]. Over the last decade, multiple trials have demonstrated the safety of ascorbic acid alone or in combination with chemotherapy [8]. Some studies suggest that ascorbate could even alleviate the adverse effects of chemotherapy without sacrificing treatment efficacy and improve quality of life for cancer patients [9, 10]. Additionally, pharmacological ascorbate has shown *in vivo* efficacy in a variety of cancers as a monotherapy and in combination with existing cancer drugs, including agents known to cause DNA damage, such as cisplatin [11, 12, 13]. While there is still uncertainty regarding precisely how ascorbate exerts anticancer effects, one established mechanism suggests high‐dose ascorbate generates reactive oxygen species (ROS) [14, 15, 16]. The propensity for ascorbate to generate ROS and subsequent DNA damage makes it an intriguing agent to pair with drugs that inhibit DNA damage repair, such as Poly(ADP‐ribose) Polymerase (PARP) inhibitors which are FDA approved for a subtype of PCa patients whose tumors are defective in homologous recombination DNA repair.

PARP refers to a family of proteins that provide diverse enzymatic functions [17, 18]. PARP‐1 is the most abundant member of the PARP family and is intimately involved in the base excision repair (BER) process of DNA repair [19]. BER is activated in response to DNA damage and enables repair of single‐stranded breaks (SSB). Inhibition of PARP‐1 compromises BER and causes accumulation of SSB which subsequently become double‐stranded breaks (DSB) during DNA replication. Briefly, PARP inhibitors (PARPi) function as NAD+ mimetics that block the active site of PARP‐1 and PARP‐2, preventing the signaling cascade needed to repair SSBs. DSB are preferentially repaired by the high‐fidelity homologous recombination (HR) pathway which includes two well‐known tumor suppressors, *BRCA1* and *BRCA2*. Accumulation of DNA damage leads to genomic instability and, ultimately, cell death [20, 21, 22]. *BRCA2* mutation is a key predictive biomarker for olaparib sensitivity in patients with PCa. Thus, we would anticipate that BRCA2 mutants should respond better than wild‐type in the combination therapy, similar to PARPi as a monotherapy. PARP inhibitors also function to kill HR‐deficient tumor cells via PARP trapping as an additional mechanism of action. Thus, the potential for PARP inhibitors to selectively kill cells with known deficiencies in DNA damage repair (DDR) pathways has led to FDA approval in certain tumor types harboring defects in DNA damage repair, including PCa.

The PARP inhibitors, in combination with other agents, have demonstrated benefit in specific PCa populations, particularly those harboring homologous recombination deficiency (HRD). Three recent Phase III trials demonstrated efficacy in combining abiraterone with PARP inhibitors (MAGNITUDE [23, 24]: NCT0374861, niraparib; PROpel [25, 26]: NCT03732820, olaparib; TALAPRO‐2 [27, 28, 29, 30]: NCT03395197, talazoparib). In the PROpel trial, the combination of abiraterone and olaparib demonstrated clinical benefit in patients with metastatic mCRPC, with the strongest signal observed in HRD‐positive patients [25, 26]. Similarly, the MAGNITUDE trial evaluated the addition of niraparib to abiraterone and showed significant progression‐free survival (PFS) improvement in HRD‐positive patients, particularly those with BRCA1/2 mutations, but did not find benefit in the HRD‐negative cohort [23, 24]. Furthermore, the TALAPRO‐2 trial demonstrated that the combination of talazoparib and enzalutamide led to improved PFS in mCRPC patients, with the HRD‐positive subgroup showing the greatest benefit [27, 28, 29, 30]. These findings underscore the importance of biomarker‐driven patient selection for PARP inhibitor‐based therapies.

MAGNITUDE was preselected for HR defects, whereas PROpel found that there were responses to the combination of PARP inhibition and abiraterone irrespective of HR status [25]. While less than 5% of primary prostate cancers harbor mutations in HR genes [31], these mutations are elevated in mCRPC, albeit only to ~23% [32]. Importantly, mutations in HR genes are not always associated with HRD nor clinical response to PAPRi. While FDA approved for mCRPC and having shown benefit in combination with abiraterone, it is critical to combine PARP inhibition with other agents to expand the cohort of patients who might benefit from PARP inhibitor therapy. Ultimately, the combination of ascorbate, which has demonstrated tolerability and efficacy in killing cancer cells by generating DNA‐damaging ROS, and PARP inhibitors, which are known to impair a cancer cell's ability to repair DNA damage and have already demonstrated efficacy in treating advanced PCa as a monotherapy, could provide a new treatment modality for the mCRPC population.

We hypothesized that DNA‐damaging ROS generated by ascorbate would pair well with PARP inhibitors and their ability to impede DNA damage repair. Combination studies demonstrated a decrease in CRPC proliferation *in vitro* and *in vivo*. The combination of olaparib and ascorbate was so potent as to demonstrate synergy in two distinct *in vitro* models. Mechanistic studies showed a significant increase in ROS accumulation and DNA damage, supporting the initial hypothesis. These results suggest the combination of ascorbate and PARP inhibition could be an effective treatment in mCRPC.

## 2 Materials and methods

### 2.1 Cell lines, cell culture and treatment

C4‐2 and 22Rv1 cells were purchased from ATCC, authenticated by ATCC, and assayed for mycoplasma upon thawing. C4‐2 (RRID:CVCL_4782) cells are categorized as p53‐functional, express a mutated version of the androgen receptor (AR), and are therefore capable of proliferating in androgen‐deprived conditions. 22Rv1 (RRID:CVCL_1045) cells are heterozygous for p53 mutation, express a splice variant of AR known as AR‐V7, and are also capable of proliferating in androgen‐deprived conditions. All experiments were performed with mycoplasma‐free cells. Detailed media culture conditions can be found in Fig. S1A and Table S1. C4‐2 cells were cultured and maintained in Improved Minimum Essential Medium (IMEM) (Thermo Fisher Scientific, Waltham, MA, USA, 10024CV) supplemented with 5% FBS (fetal bovine serum, heat inactivated), 1% l‐glutamine (2 mmoL·L^−1^), and 1% penicillin–streptomycin (100 units·mL^−1^). RPMI and IMEM have been utilized as base media for C4‐2 and other PCa cell lines in prior studies. For the experiments in this manuscript, IMEM was selected as the primary medium for hormone‐deprivation studies (e.g., experiments using charcoal‐dextran treated serum), as it provided the most reproducible results [33, 34]. Thus, the studies in this manuscript utilized IMEM for consistency and comparison of findings for the assays conducted. 22Rv1 cells were cultured and maintained in Dulbecco's modified Eagle medium (DMEM) (Thermo Fisher Scientific, 10017CV) supplemented with 10% FBS, 1% L‐glutamine (2 mmoL·L^−1^), and 1% penicillin–streptomycin (100 units·mL^−1^). All cells were cultured at 37 °C with 5% CO_2_. For indicated experiments, the cell culture media was supplemented with sodium pyruvate (Sigma‐Aldrich, St. Louis, MO, USA, P2256‐5G made at 100 mm stock stored at 4 °C), iron nitrate (Sigma‐Aldrich 216 828‐100G made at 50 mm stock stored at 4 °C), or iron chloride (Sigma‐Aldrich 236 489‐5G made at 50 mm stock stored at 4 °C). Treatment then proceeded with Sodium L‐ascorbate (Sigma‐Aldrich A4034‐100G made at 100 mm stock and stored at 4 °C), olaparib (Selleck S1060 made at 100 mm stock and stored at ‐20 °C), niraparib (AdooQ BioScience A11026 made at 100 mm stock and stored at −20 °C), and/or talazoparib (Selleck S7048 made at 10 mm stock and stored at −80 °C) depending on the experiment. For experiments in which cells were plated and treated in the alternate cell culture medium (C4‐2 in DMEM and 22Rv1 in IMEM), cells were initially thawed into their original medium and passaged 3 times in the alternate medium to ‘condition’ them prior to use for experiments.

### 2.2 Proliferation assays

Cell viability was determined by double‐stranded DNA assay. Briefly, equivalent densities of C4‐2 or 22Rv1 cells were seeded in their respective media on clear, Poly‐l‐lysine‐coated 96‐well plates and allowed to adhere overnight. Cells were then pretreated and/or treated with the reagents previously described. After treatment, the media was discarded, cells were gently washed in PBS several times and cells were lysed in 100 μL of dH_2_O for 1 h at 37 °C. Cells were then incubated with the Quanti‐IT PicoGreen dsDNA reagent (Invitrogen, Carlsbad, CA, USA, P7581) per manufacturer's instructions. Data were collected using a BioTek Synergy HT plate reader. Cells were compared to Day 0 for normalization. For synergy calculations, the compusyn software from https://www.combosyn.com/ was utilized following threshold and analyses guidelines as detailed by the software program.

### 2.3 Metabolic assays

#### 2.3.1 Pyruvate assay

Equivalent densities of C4‐2 or 22Rv1 cells were seeded on Poly‐L‐lysine coated 96‐well plates and allowed to adhere overnight. At indicated time points during treatment, cell culture media were collected, and pyruvate concentration was assessed using the Pyruvate Colorimetric Assay Kit (BioVision, Milpitas, CA, USA, K609‐100) per manufacturer's instructions. Absorbance was detected using a BioTek Synergy HT plate reader.

#### 2.3.2 ATP assay

Equivalent densities of C4‐2 or 22Rv1 cells were seeded on Poly‐L‐lysine coated, white‐walled 96‐well plates (Sigma‐Aldrich CLS3610‐48EA) and allowed to adhere overnight. At indicated time points during treatment, intracellular ATP levels were assessed using the CellTiter‐Glo 2.0 Cell Viability Assay (Promega Corporation, Madison, WI, USA, G9242) per the manufacturer's recommendations. Luminescence was detected using a BioTek Synergy HT plate reader.

#### 2.3.3 ROS assay

Equivalent densities of C4‐2 or 22Rv1 cells were seeded in their respective media on Poly‐L‐lysine coated, white‐walled 96‐well plates (Sigma‐Aldrich CLS3610‐48EA) and allowed to adhere overnight. Cells were then pretreated and/or treated with the reagents previously described. After treatment, ROS levels were assessed using the ROS‐Glo H_2_O_2_ Assay (Promega G8821) per manufacturer's instructions. Briefly, cells were pre‐incubated with the ROS substrate for 2 h at 37 °C prior to analysis at which point the detection reagent was added for 20 min at room temperature. ROS levels were detected by quantifying luminescence using a BioTek Synergy HT plate reader.

### 2.4 Immunofluorescence (IF)

Immunofluorescence (IF) experiments were performed as detailed previously [33]. Briefly, equivalent densities of C4‐2 or 22Rv1 cells were seeded on poly‐lysine coated coverslips in 6‐well plates and allowed to adhere overnight. After treatment, coverslips were washed gently in PBS and fixed for 20 min with 3.7% formaldehyde at room temperature. Cells were stained using γH2AX phospho‐S139 (EMD Millipore, Billerica, MA, USA, 16‐202A) at a 1:500 dilution. Foci were imaged utilizing Dr. Elda Grabocka's Zeiss Cell Discoverer Confocal Microscope at 40× magnification with at least 5 fields for each replicate. Fiji image software was utilized to quantify foci formation per cell and compared to control samples.

### 2.5 Immunoblotting

C4‐2 and 22Rv1 cells were plated at equal densities in their respective media on Poly‐L‐lysine coated 10 cm^2^ plates. Generation of cell lysates was described previously [34]. Briefly, 40–50 μg of lysate was resolved by SDS/PAGE, transferred to nitrile membrane, and analyzed using the following antibodies: P21 (1:1000, Abcam, Cambridge, MA, USA, ab109520), phospho‐CDC2 (Tyr15) (1:1500, Cell Signaling Technology, Danvers, MA, USA, 9111S), CDC2 p34 (1:200, Santa Cruz Biotechnology, Dallas, TX, USA, Sc‐54), and Vinculin (1:1000, Sigma‐Aldrich V9264‐200uL).

### 2.6 Generation of xenografts

C4‐2 cells were cultured and lifted from plates by trypsinization then resuspended in 100 mL of 50% Matrigel (BD Biosciences, San Jose, CA, USA) and saline mixture followed by subcutaneous injection in two separate groups of castrated and noncastrated athymic, male, nude NOD/SCID mice (age at least 6 weeks old). These mice were purchased from Charles Rivers, Inc. Once tumors reached 100 mm^3^ in size, mice were triaged into four separate treatment groups (*n* = 5 mice per group). Mice were treated daily via intraperitoneal (IP) injections of either vehicle control (0.09% saline), ascorbate (4 g·kg^−1^), olaparib (50 mg·kg^−1^), or a combination of both. Treatment continued until humane endpoints were reached, defined as tumor size exceeding 1000 mm^3^. Most animals were treated for 4 weeks, while some cohorts with slower tumor progression were treated for up to 10 weeks. Tumor volume was measured every other day using calipers, and body weights were monitored weekly for the duration of the experiment. When tumor sizes reached 1000 mm^3^ in size mice were euthanized and their tumors harvested and fixed in 4% formalin in preparation for sectioning, mice were housed in standard conditions. All animal work was done in accordance with and in compliance with the regulations set forth by the Institutional Animal Care and Use Committee (IACUC) at Thomas Jefferson University and SKCC (protocol #00936 under PI – Dr Karen E Knudsen). Briefly, mice were housed in ventilated cages under standard conditions. This consisted of 12‐h light/dark cycles, 20–26 °C temperature, 40–60% humidity, and with access to chow and autoclaved water. Standard nesting material was provided. All procedures were performed under aseptic conditions, and animals were monitored daily for health and welfare.

### 2.7 Immunohistochemistry (IHC)

For histological analysis from xenograft tissue, formalin‐fixed, paraffin‐embedded (FFPE) sections were stained with hematoxylin and eosin (H&E), Ki67, and p21 (Cell Signaling S947S, 1:50 dilution) as previously described [34]. Xenograft tissues were scored using an Aperio microscope and software. Briefly, 20× and 40× magnification images were taken and positive staining was assayed for the full section of tissue to be reported from the whole slide scan.

### 2.8 Statistical analysis

All experiments were performed in technical triplicate with at least 3 biological replicates per condition. Data are displayed as mean ± standard error of the mean (SEM). Statistical significance (*P* < 0.05) was determined using Student's *t*‐test.

## 3 Results

### 3.1 Ascorbate alters energy dynamics of CRPC cells and generates ROS resulting in DNA damage

Ascorbate toxicity has been demonstrated in several different types of cancer cells; however, the evidence supporting its use in advanced PCa is minimal [8, 9, 10, 11, 12, 13]. It has been well established that pharmacological ascorbate generates DNA‐damaging ROS; however, recent data in pancreatic cancer models also suggest that ascorbate can alter cellular bioenergetics [35]. No studies exploring bioenergetics have been performed in PCa. The antitumor effects of ascorbate were impacted by both iron and pyruvate concentration in the culture media (Fig. S1B). Pyruvate can act as an antioxidant in cancer cells, particularly under conditions of oxidative stress [36, 37]. Importantly, manipulating these culture media parameters did not impact cancer cell growth in the absence of ascorbate (Fig. S1B,C). This study will be the first to identify the novel role of ascorbate enhancing PCa therapeutics.

**Fig. 1:**
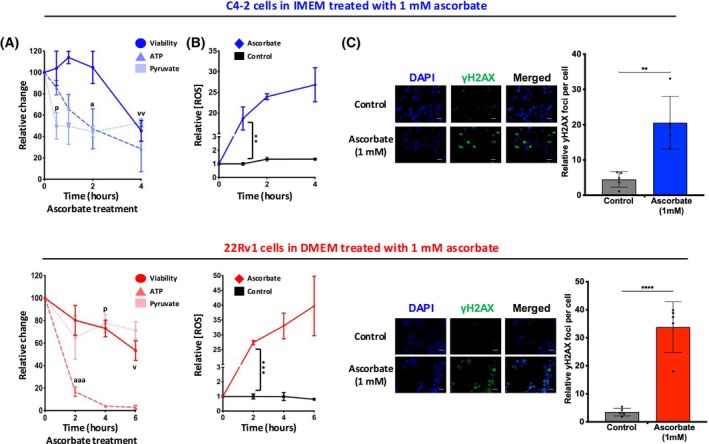
Ascorbate alters energy mechanics of castration‐resistant prostate cancer (CRPC) cells and generates reactive oxygen species (ROS) resulting in DNA damage. (A) Metabolic profiling of CRPC cells with ascorbate treatment over time. Intracellular ATP and pyruvate levels were quantified in C4‐2 and 22Rv1 cells following ascorbate treatment. C4‐2 cells (top) were conditioned in IMEM, seeded onto 96‐well plates and allowed to settle overnight. Wells were treated with 1 mm ascorbate, after which levels of extracellular pyruvate (square), intracellular ATP (triangle), and cell viability (circle) were assessed at the indicated time points. 22Rv1 cells (bottom) were plated in DMEM, treated, and analyzed as described for C4‐2 cells. (B) C4‐2 cells (top) and 22Rv1 (bottom) were plated as described in (A), and levels of ROS were assessed at the indicated time points. These findings demonstrate that ascorbate induces metabolic disruption and ROS generation, consistent with proposed pro‐oxidant mechanisms relevant to clinical translation. (C) C4‐2 cells (top) and 22Rv1 cells (bottom) were plated as described in (A). C4‐2 cells (2 h) and 22Rv1 cells (4 h) were treated with 1 mm ascorbate and assessed for formation of γH2AX foci as a surrogate marker for DNA damage. Scale bar 250 μm. Data are depicted as mean relative to vehicle control ± SEM (standard error of the mean) of at least three independent biological replicates. Statistical significance was determined by Student's *t*‐test. ** denotes *P* < 0.01 compared to untreated control, *** denotes *P* < 0.001 compared to untreated control, **** denotes *P* < 0.001 compared to untreated control, *a* denotes *P* < 0.01 compared to ATP levels at 0 h treatment, *aaa* denotes *P* < 0.001 compared to ATP levels at 0 h treatment, *P* denotes *P* < 0.05 compared to pyruvate levels at 0‐h treatment, *v* denotes *P* < 0.05 compared to viability at 0‐h treatment, *vv* denotes *P* < 0.01 compared to viability at 0‐h treatment.

To assess the potential antitumor effects of ascorbate, CPRC models, C4‐2 and 22Rv1 cells, were conditioned as described in Materials & Methods and treated with 1 mm ascorbate for up to 6 h. The base dosage of ascorbate utilized in this study was determined through a series of preliminary growth assays, evaluating increasing concentrations for biological relevance and toxicity. The selected concentration reflects physiologically relevant plasma levels achievable through intravenous administration in humans, as reported in prior clinical studies. Additionally, this concentration was nontoxic as a monotherapy in the C4‐2 models and permitted further investigation of combinatorial effects without confounding toxicity. The extracellular concentration of pyruvate was assayed at several time points during treatment. Within 30 min of initiating treatment with ascorbate, the concentration of pyruvate decreased by approximately 50% for C4‐2 cells compared to untreated control (*P* = 0.020; Fig. 1A, top). Similarly, within 4 h of treatment with ascorbate, 22Rv1 cells saw a greater than 20% reduction in pyruvate concentration (*P* = 0.048, Fig. 1A, bottom) compared to untreated control. Additionally, within 2 h of ascorbate administration, the amount of intracellular ATP decreased by over 50% (*P* = 0.049; Fig. 1A, top) or 80% in C4‐2 and 22Rv1 cells (*P* = 7.83e‐4; Fig. 1A, bottom), respectively. These findings were associated with a 55% decrease in the percentage of surviving C4‐2 cells within 4 h compared to untreated control (*P* = 0.010; Fig. 1A, top) and by nearly 50% decrease in surviving 22Rv1 cells within 6 h (*P* = 0.011, Fig. 1A, bottom). These findings indicate the antitumor effects of ascorbate are associated with altered cellular metabolism that precedes reduction in cancer cell viability.

The combined impact of these data is to establish that treatment with high‐dose ascorbate affects CRPC cell energy mechanics as evidenced by a decrease in extracellular pyruvate, intracellular ATP, and ultimately cell viability. These observations are explicable by considering ascorbate‐induced ROS and the effects of ROS on cancer cell metabolism. Cancer cells have constitutively higher levels of pro‐tumorigenic ROS [38] than nontransformed cells; however, excess ROS can damage mitochondrial DNA as well as key proteins in the electron transport chain necessary for generating ATP by oxidative phosphorylation [39]. Additionally, an excess of ROS forces cancer cells to shift substrates from the ATP‐generating Krebs cycle into the Pentose Phosphate pathway which generates NADPH, a key reducing agent functioning to restore glutathione into its reduced antioxidant form [40]. Thus, there is a dual mechanism involving the impairment of oxidative phosphorylation and glycolysis by which ascorbate‐mediated ROS could rapidly deplete cellular ATP. To determine whether elevated ROS was associated with the observed altered cancer cell metabolism and subsequent reduction in cancer cell viability, it was necessary to establish the timing and degree to which ascorbate generates ROS in CRPC models.

A time‐dependent accumulation of ROS was observed after 60 min of ascorbate treatment with a greater than 18‐fold increase in measured ROS for C4‐2 cells treated with ascorbate compared to untreated control (*P* = 0.008, Fig. 1B, top) and a 27‐fold increase observed in 22Rv1 cells (*P* = 2.64 × 10^−4^, Fig. 1B, bottom) within 2 h of ascorbate treatment. Immunofluorescent staining for yH2AX showed a 5.5‐fold increase in yH2AX foci in C4‐2 cells treated with ascorbate for 2 h compared to untreated control (*P* = 0.031, Fig. 1C, top) and a 9‐fold increase in 22Rv1 cells treated with ascorbate for 4 h (*P* = 0.027, Fig. 1C, bottom). Thus, these data suggest that treatment with high‐dose ascorbate generates cytotoxic levels of ROS that can impair cell metabolism and generate DNA damage. Whether these mechanisms are independent of each other or related is unclear; however, these data indicate that ascorbate monotherapy can kill CRPC cells at physiologically achievable doses making it an attractive therapeutic option and a candidate for combination with other treatments with varying mechanisms of action. Given the observed increase in DNA damage associated with ascorbate monotherapy, it was hypothesized that therapeutic benefit could be achieved by adding an agent which prevents the repair of DNA damage, such as a PARP inhibitor.

### 3.2 Treatment with ascorbate and olaparib synergistically inhibits CRPC cell proliferation *in vitro* by generating DNA damage

**Fig. 2:**
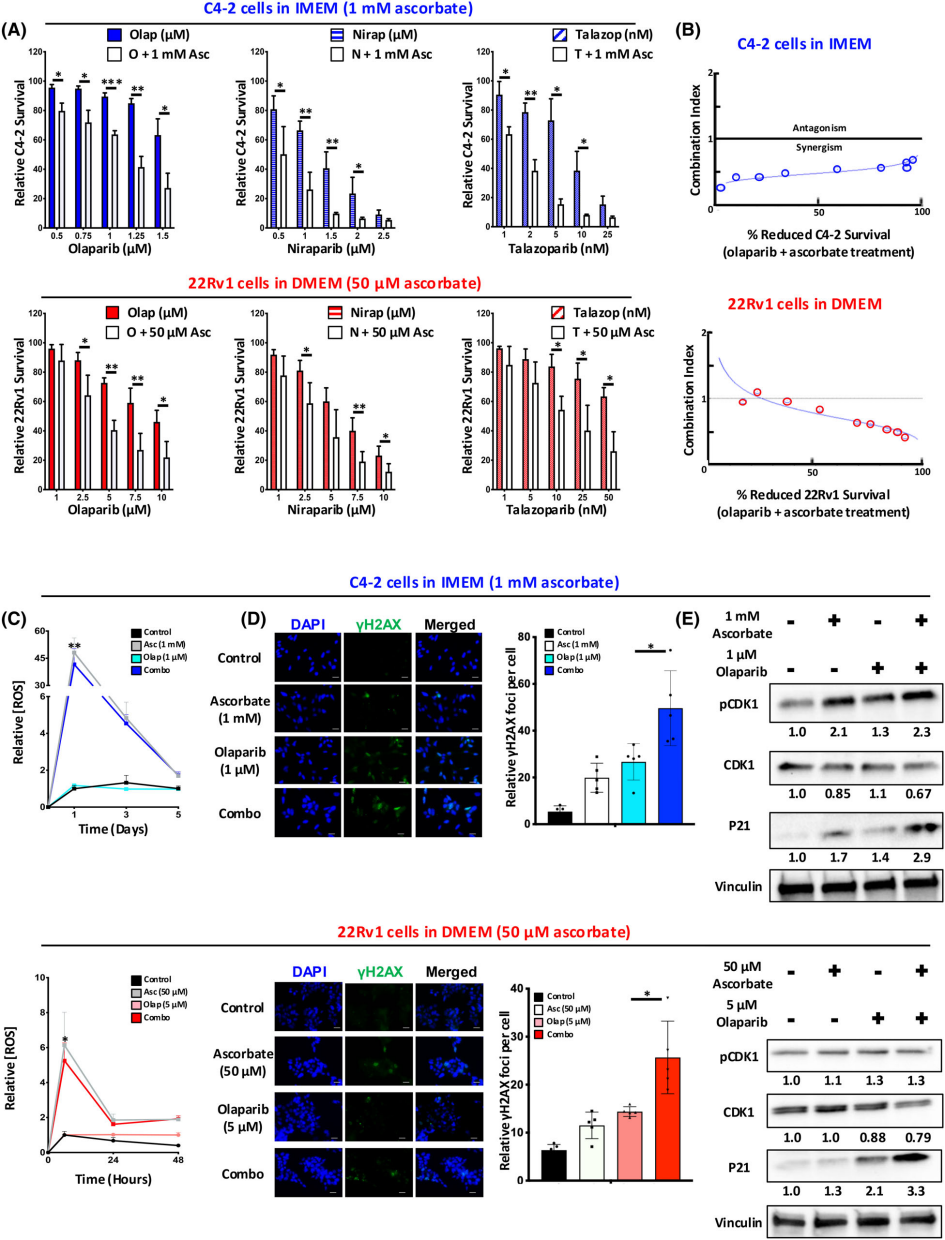
The combination of ascorbate and olaparib synergistically inhibits castration‐resistant prostate cancer (CRPC) cell proliferation *in vitro* by generating DNA damage. (A) Combination effects of ascorbate and PARP inhibition on CRPC cell growth. Equivalent densities of C4‐2 cells (top) and 22Rv1 cells (bottom) were seeded on 96‐well plates in IMEM and DMEM, respectively. Cells were treated with a combination of ascorbate and either olaparib (left), niraparib (center), or talazoparib (right) at the indicated concentration for 5 days. Cell survival was assessed using the PicoGreen assay. (B) CompuSyn was used to generate a combination index (CI) for the combination of ascorbate and olaparib in C4‐2 cells (top) and 22Rv1 cells (bottom). CI > 1 denotes antagonism, CI = 1 denotes an additive effect, CI < 1 denotes synergism. The concentration range of olaparib is 0.5–3 μm (C4‐2 cells) and 1–20 μm (22Rv1 cells). The concentration range of ascorbate is 1–2.5 mm (C4‐2 cells) and 25–150 μm (22Rv1 cells). (C) Equivalent densities of C4‐2 cells (top) or 22Rv1 cells (bottom) were seeded on a 96‐well plate and treated with the indicated amount of ascorbate and olaparib alone and in combination. The levels of reactive oxygen species (ROS) were assayed at the indicated time points. (D) Equivalent densities of C4‐2 cells (top) or 22Rv1 cells (bottom) were seeded on 6‐well plates and treated with the indicated amount of ascorbate and olaparib alone and in combination for 3 days. Cells were assessed for formation of γH2AX foci as a surrogate marker for DNA damage. Scale bar 250 μm. (E) C4‐2 cells (top) or 22Rv1 cells (bottom) were seeded on 10 cm^2^ plates and treated with the indicated amount of ascorbate and olaparib alone and in combination for 3 days. Culture media and attached cells were collected for western blot analysis of proteins associated with G2/M checkpoint. Data are depicted as mean relative cell survival (compared to vehicle control) ± SEM of at least three independent biological replicates. Statistical significance was determined by Student's *t*‐test. * denotes *P* < 0.05, ** denotes *P* < 0.01, *** denotes *P* < 0.001.

Three different PARP inhibitors, olaparib (Fig. 2A, left), Niraparib (Fig. 2A, middle), and Talazoparib (Fig. 2A, right) were assessed for efficacy in treating C4‐2 cells (Fig. 2A, top) and 22Rv1 cells (Fig. 2A, bottom). All three PARP inhibitors demonstrated a dose‐dependent ability to inhibit CRPC cell proliferation as monotherapy which was not impacted by pyruvate concentration (Fig. S2A,B). The base media of IMEM and DMEM have different levels of pyruvate and iron, which are known to scavenge ROS generated by ascorbate. IMEM contains 1 mm pyruvate and 2 μm iron, while DMEM contains no pyruvate and 250 nm iron. These differences in media composition significantly impact the oxidative state of the cells and their sensitivity to ascorbate. To ensure equal comparison, we accounted for these differences by using media‐specific conditions for experiments assessing ascorbate sensitivity in C4‐2 and 22Rv1 cells. Further testing is required to assess the broader biological implications of media composition on ROS‐driven cytotoxicity.

For this study, cell viability was determined by double‐stranded DNA assay and not MTS assay to allow for analysis of treatment on cell growth. Intriguingly, the addition of a nonlethal dose of ascorbate significantly augmented the toxicity of all three PARP inhibitors in both CRPC models. Given these promising results, the possibility of synergy between ascorbate and PARP inhibitors was investigated. Olaparib, being the most clinically advanced of the three PARP inhibitors tested, was used to generate additional growth curves with ascorbate. Data were analyzed using the program CompuSyn to develop a combination index for the combination of olaparib and ascorbate in both C4‐2 cells (Fig. 2B, top) and 22Rv1 cells (Fig. 2B, bottom). For both CRPC models, most data points generated fall into the region where the combination index is less than one, suggesting a strongly synergistic relationship between olaparib and ascorbate *in vitro*. With higher potency, talazoparib and niraparib did not meet the threshold for synergistic effects with ascorbate, while olaparib had a synergistic effect. Testing talazoparib at lower concentrations also failed to demonstrate synergy, suggesting that the robust single‐agent efficacy of talazoparib may mask potential combinatory effects. These findings underscore the unique profile of olaparib in demonstrating a synergistic relationship with ascorbate under the tested conditions.

Importantly, rescue experiments revealed that the addition of pyruvate significantly reversed the antigrowth effects of ascorbate in combination with olaparib and niraparib (Fig. S2C). This finding highlights the critical role of ROS generation in mediating the synergy between ascorbate and PARP inhibition, as pyruvate acts as a ROS scavenger to mitigate ascorbate's cytotoxic effects. Consistent with this mechanism, we also observed that pyruvate suppressed ascorbate‐induced ROS production (Fig. S2D). To further confirm the role of ROS in ascorbate‐mediated growth inhibition, we tested the impact of an independent antioxidant, glutathione. Similar to pyruvate, glutathione effectively rescued cell viability in ascorbate‐treated cells, reinforcing the role of oxidative stress in the observed cytotoxicity (Fig. S2E). These findings underscore the importance of ROS generation in the therapeutic effects of ascorbate and its interaction with PARP inhibitors.

In addition to PARP inhibitors, we evaluated the combinatorial effects of ascorbate with other DNA‐damaging agents, including a taxane (e.g., paclitaxel) and doxorubicin, in CRPC models (C4‐2 and 22Rv1 cells). Ascorbate enhanced the growth inhibitory effects of these chemotherapies, consistent with its role in promoting oxidative stress and impairing DNA repair mechanisms (Fig. S2F,G), demonstrating the independent and combined contributions of these treatments. These results further support the broad potential of ascorbate in combination with DNA‐damaging therapeutics for advanced PCa.

Given the capacity of ascorbate to generate ROS as a single agent, the impact of PARPi on ascorbate‐driven ROS generation was determined. Even the nonlethal dose of ascorbate was associated with significant ROS accumulation in C4‐2 cells, a 48‐fold increase compared to untreated control after 24 h (*P* = 0.009, Fig. 2C, top), and 22Rv1 cells, a 6‐fold increase compared to untreated control after 6 h (*P* = 0.041, Fig. 2C, bottom). Of note, the ROS level appeared to peak within 24 h and rapidly decline, mirroring the rapid metabolism of ascorbate seen in human subjects. Furthermore, the addition of olaparib did not impact the extent or duration of ROS accumulation.

Utilizing yH2AX as a marker for DNA double‐strand breaks, C4‐2 cells treated with the combination of ascorbate and olaparib generated 11‐fold more yH2AX foci relative to untreated control compared to a 6‐fold increase by olaparib alone (*P* = 0.017, Fig. 2D top). A similar pattern was observed in 22Rv1 cells where a 2.5‐fold increase was generated by the combination treatment relative to untreated control compared to a 1.7‐fold increase by olaparib alone (*P* = 0.019, Fig. 2D bottom). Given the instability of ascorbate under *in vitro* conditions, it was added as a single dose at the start of the experiment. This approach maintains clinical relevance by reflecting a feasible dosing regimen, as continuous or repeated ascorbate dosing would not be practical in a clinical setting. The end time points were selected to assess the sustained effects of a single ascorbate dose, particularly in combination with PARP inhibitors, to capture downstream effects such as ROS‐induced DNA damage (γH2AX) and changes in cell viability. Interpreting the ROS and yH2AX data together suggest that combination treatment does not generate additional ROS compared to monotherapy; however, increased accumulation of DNA damage is observed. This is consistent with the hypothesis that PARP inhibition, while having no effect on the presence of ROS, would correlate with a delay in the repair of DNA damage generated by the presence of ROS. While these findings suggest differences in the signaling axis across CRPC models, it is important to acknowledge that 22Rv1 and C4‐2 cells differ in several biological characteristics in addition to their *TP53* status. As such, the results presented here cannot definitively attribute the observed effects to *TP53* status alone. Future studies utilizing isogenic cell lines with *TP53* inactivation will be necessary to rigorously assess the specific impact of *TP53* on this signaling pathway. Ultimately, ascorbate‐induced ROS generation leads to an elevation in DNA double‐strand breaks, and the addition of PARPi to ascorbate results in a further accrual of DNA damage.

To examine the impact of the tested therapies on DNA repair factors, protein expression was assessed with combination therapy in CRPC models. Western blot analysis for C4‐2 cells treated with ascorbate and olaparib alone or in combination showed a significant increase in p21 protein concentration and activity, as evidenced by an increase in phosphorylated CDK1, compared to either monotherapy or untreated control (Fig. 2E, top). A similar pattern was seen in 22Rv1 cells (Fig. 2E, bottom). p21 expression is increased in response to DNA damage and one of its many functions is to inhibit CDK1 by phosphorylation to halt the cell cycle at the G2/M checkpoint and allow for repair of DNA damage [41]. In response to ascorbate plus olaparib treatment, both C4‐2 and 22Rv1 cells showed an increase in p21 expression, indicating cell cycle arrest. However, pCHK1 activation was more prominent in C4‐2 cells, while minimal alterations in pCDK1 were observed in 22Rv1 cells. This difference could stem from inherent differences in cell cycle control mechanisms between the two cell lines, including variations in androgen receptor (AR) signaling and baseline CDK1 activity. C4‐2 cells, which are AR‐positive and castration‐resistant, exhibit more pronounced alterations in pCHK1 activation and cell cycle regulation in response to ascorbate plus olaparib compared to 22Rv1 cells. These findings suggest that differences in signaling pathways and cell cycle regulation may contribute to the observed variability in treatment response between the cell lines. Taken together, these data suggest that the combination of ascorbate and olaparib inhibits CRPC cell proliferation synergistically *in vitro* by generating DNA damage.

### 3.3 The combination of olaparib and ascorbate slows tumor growth *in vivo*

**Fig. 3:**
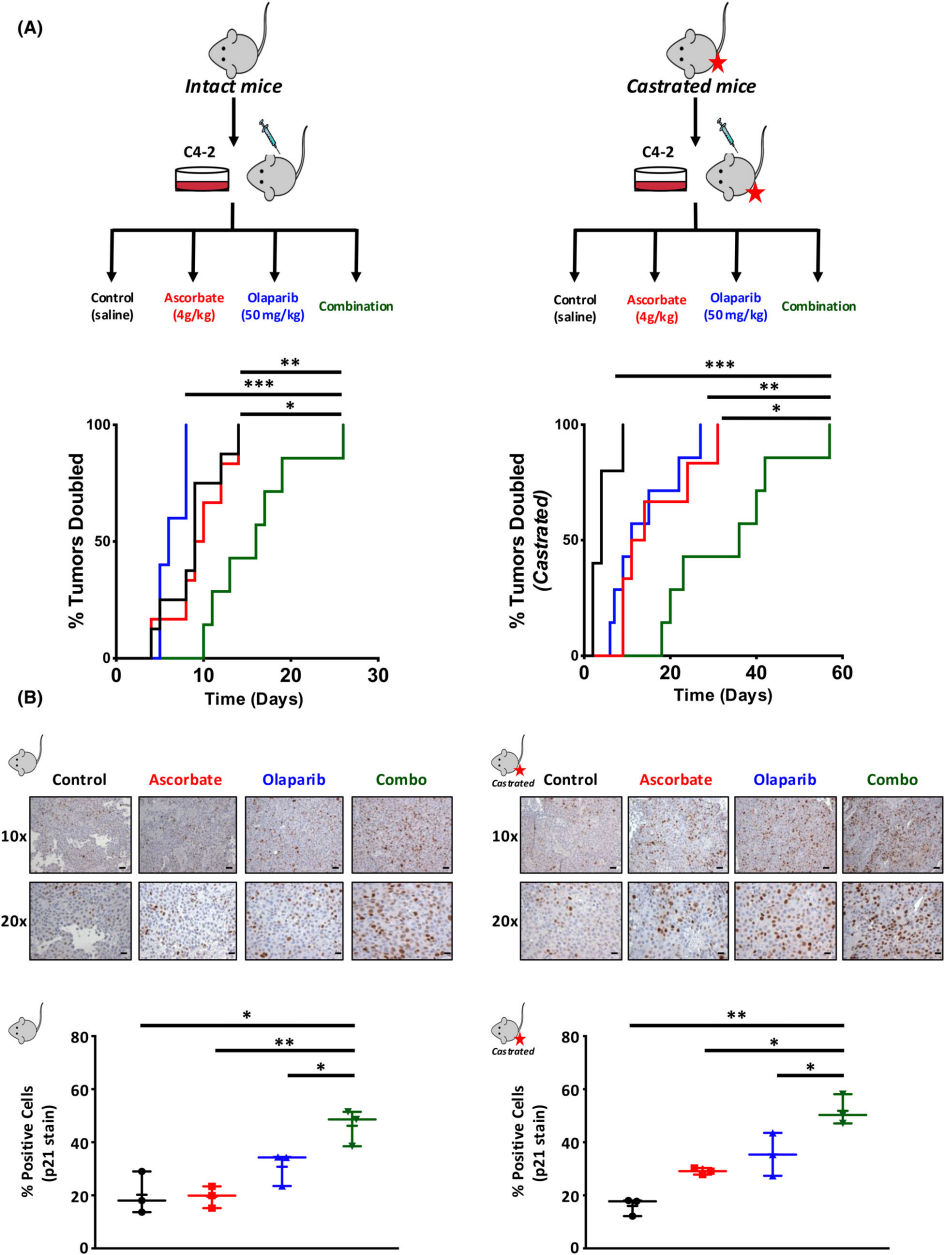
The combination of olaparib and ascorbate slows tumor growth *in vivo*. (A, B) *In vivo* effects of ascorbate and olaparib in castration‐resistant prostate cancer (CRPC) xenograft models. (A) C4‐2 xenografts were generated in either noncastrated (left) or castrated (right) NOD/SCID mice. Mice that developed tumors were randomly assigned into cohorts receiving daily IP injections of normal saline, 50 mg·kg^−1^ olaparib, 4 g·kg^−1^ ascorbate or a combination of 50 mg·kg^−1^ olaparib and 4 g·kg^−1^ ascorbate (*n* = 5 mice per group). Tumor volume was measured by calipers and calculated by *V*_tumor_ = (short distance)^2^ × long distance × 0.5236. Statistical significance was determined by Log‐rank (Mantel–Cox) test. Line graphs represent individual tumor volumes, and % tumors doubled are shown. Statistically differences are observed in the monotherapies and combination therapy as denoted with the asterisks. (B) Harvested tumors underwent immunohistochemistry (IHC) analysis for P21. Representative images are shown at 10× and 20×, and quantitative analysis is shown below. Scale bars are 125 μm and 250 μm, respectively. Three slides, each from a different tumor, were chosen from each treatment group, and five randomly chosen fields from each slide were photographed and scored. Data depicted represent mean % positive cells. Data are depicted as mean relative to vehicle control ± SEM (standard error of the mean). All panels display data for intact hosts on the left and castrated hosts on the right to maintain consistency. Tumor doubling times were calculated based on exponential growth models, and statistical comparisons between treatment groups were performed using ANOVA with Tukey's *post hoc* test. * denotes *P* < 0.05, ** denotes *P* < 0.01, and *** denotes *P* < 0.001.

Given the promising *in vitro* results generated by combining ascorbate and olaparib, the impact of this treatment *in vivo* was next investigated. Xenografts were generated by injecting C4‐2 cells into both flanks of two cohorts, one cohort of castrated and one cohort of noncastrated, immunocompromised mice. The mice were then randomly assigned to receive daily IP injections of saline, ascorbate alone, olaparib alone, or the combination of olaparib and ascorbate. Tumor growth was measured three times weekly. Mice treated with the combination of ascorbate and olaparib showed significantly increased tumor doubling time compared to mice treated with olaparib alone (*P* = 5.00 × 10^−4^) or ascorbate alone (*P* = 0.015) in both noncastrated (Fig. 3A, left) and castrated conditions (*P* = 0.007 and *P* = 0.022, respectively, Fig. 3A, right). Body weight data for the full treatment duration of the *in vivo* studies for both castrated and noncastrated experiments confirm that the mice weight loss did not exceed 20% before humane endpoints were reached (Fig. S3A,B). Tumor doubling time analysis revealed differences in the efficacy of ascorbate and olaparib monotherapies between intact and castrated hosts. In the castrated host, monotherapies exhibited moderate effects on tumor growth, with ascorbate and olaparib demonstrating greater efficacy than in the intact host. This observation may reflect the role of androgen deprivation in altering tumor biology, potentially enhancing the tumor's susceptibility to oxidative stress and DNA damage repair modulation. Further studies are warranted to investigate these interactions in greater detail. Upon reaching the endpoint, tumors were harvested, and tissue underwent IHC staining for p21. Tumors from mice treated with the combination of ascorbate and olaparib showed significantly increased percent positive cells compared to mice treated with olaparib alone (*P* = 0.050) or ascorbate alone (*P* = 0.008) in both noncastrated (Fig. 3B, left) and castrated conditions (*P* = 0.050 and *P* = 0.016, respectively, Fig. 3B, right) with minimal impact on animal weight and no impact on Ki67 (Fig. S3C–E). Interestingly, the observed reduction in tumor volume may result from mechanisms other than direct suppression of proliferation, such as increased apoptosis, reduced vascularization, or alterations in the tumor microenvironment. These data suggest the combination of ascorbate and olaparib slows tumor growth *in vivo*, highlighting a potential novel combinatorial therapeutic regimen to improve patient outcome. However, further investigation is warranted to address the limitations in existing clinical data.

Currently, there are five actively recruiting trials specifically examining ascorbate for cancer treatment, all categorized as Phase 2. Although limited, human data appear promising. A recent study explored the combination of PARP inhibitors (Olaparib, Niraparib, or Talazoparib) with ascorbate in eight patients with advanced malignancies [42]. Notably, five patients achieved a partial response, and three achieved a complete response, with no reported grade 3 toxicity. The preclinical data presented herein served as the foundation for a clinical trial (NCT05501548) investigating the combination of ascorbate with olaparib in mCRPC patients. This trial was unfortunately terminated by the IRB due to low accrual, with only 4 of the planned 15 patients recruited. Importantly, these and additional ongoing clinical investigations aim to bridge the gap between preclinical promise and definitive clinical efficacy of ascorbate‐based cancer therapies.

## 4 Discussion

As discussed in the introduction, there is a high prevalence of DDR alterations in PCa, and these are associated with high‐grade histology and metastatic disease [43]. Multiple clinical trials have shown that PARP inhibition in DDR‐defective mCRPC is associated with improved progression‐free survival (PFS) [44]. Two PARP inhibitors have received FDA approval for use in mCRPC, olaparib based on an increase in PFS observed in the PROfound trial and rucaparib based on improved radiographic and prostate‐specific antigen response in the TRITON‐2 trial; similar studies are underway for talazoparib and niraparib [45]. A key limiting factor in the use of PARP inhibitors in mCRPC is that some trials indicate the necessity of DDR alterations to see efficacy. Importantly, several studies have shown that DDR inactivation is necessary to see clinical benefit from a PARP inhibitor as monotherapy. For instance, olaparib is indicated for patients with BRCA1/2 or other homologous recombination repair (HRR) gene alterations who have progressed after a novel hormonal agent, while rucaparib is approved for patients with BRCA1/2 mutations who have received androgen receptor‐directed therapy and taxane chemotherapy. While PCa is associated with DDR alterations, particularly in advanced disease, it is important to note that other cancers, such as high‐grade serous ovarian cancer, also exhibit a significant prevalence of DDR defects. Importantly, such mutations are still only present in a minority of mCRPC cases. For example, in the PROfound trial, only 28% of the screened population had a qualifying mutation [46]. This suggests that broader application of PARP inhibitors will likely require combination with other cancer therapies. Currently, there are multiple clinical trials studying the potential benefits of treating PCa by combining PARP inhibition with various therapies including ADT, immune checkpoint inhibitors, kinase inhibitors, and radiation, with the latter combination attempting to capitalize on the ability of radiation therapy to induce DNA damage [47].

The therapeutic implication of combining PARP inhibitors with an agent that generates DNA damage was the impetus for choosing ascorbate in the combination studies presented here. There are many DNA‐damaging agents from which to choose, and ascorbate was selected for several reasons including relative affordability compared to other chemotherapy agents, a favorable tolerability profile, and a growing body of evidence showing efficacy in treating a variety of cancers as monotherapy and in combination with standard of care chemotherapy and radiation treatments [8, 9, 10, 11, 12, 13]. The proposed mechanism by which ascorbate and PARP inhibition might prove effective in CRPC was based upon the tendency for ascorbate to generate ROS when dosed at high levels; ROS accumulation generates DNA damage which PARP inhibition would augment and perpetuate leading to CRPC cell death. The data presented here support this mechanism as ascorbate was shown to generate ROS *in vitro* and the combination of PARP inhibition and ascorbate produced a statistically significant increase in DNA damage as measured by γH2AX foci formation *in vitro*. It seems that ascorbate, as a DNA‐damaging agent, pairs well with PARP inhibitors.

Additional *in vitro* assays using ascorbate as monotherapy demonstrated a time‐dependent decrease in pyruvate and ATP levels subsequently followed by a significant decrease in CRPC cell proliferation. These results correlate with *in vitro* data derived from experiments in breast cancer that describe an ascorbate‐induced ‘energy catastrophe’ evidenced by rapid depletion of ATP and cell death [48]. While these results were generated in the absence of any PARP inhibition, other studies have produced evidence that, in ATP‐deficient situations, PARP enzymes generate ATP needed to properly execute BER [49]. If true, then PARP inhibition could augment the depletion of ATP that results from ascorbate treatment. This suggests the mechanism driving the synergy between these two agents may be more intricate than simply ascorbate inducing DNA damage and PARP inhibition impeding the repair process.

Further literature review sheds light on yet another facet that may influence the interaction between ascorbate and PARP inhibitors. It is well established that ascorbate is an essential cofactor for a variety of enzymes known as dioxygenases. One such family of enzymes, the ten‐eleven translocation (TET) methyl cytosine dioxygenases, catalyze the hydroxylation of methylated cytosine bases in DNA, one of the initial steps in DNA demethylation. DNA methylation is an important process by which cells can regulate the expression of specific genes, and much has been written about the significance of such epigenetic regulation in the development, progression, and treatment of cancer in general. Ascorbate's association with the TET family suggests a role in the epigenetic control of the genome and could have significant implications for cancer treatment. A plethora of *in vitro* and *in vivo* studies performed in a wide range of solid and hematological malignancies have consistently demonstrated that treatment with ascorbate, either as monotherapy or in combination with established epigenetic modifiers, significantly increases TET catalytic activity [50]. The tendency for PARP inhibitors to form complexes by directly binding PARP enzymes at sites of DNA damage makes them an intriguing target for combination with epigenetic modifiers [51]. At least one set of *in vitro* and *in vivo* experiments in ovarian cancer demonstrated that the combination of olaparib and one such epigenetic modifier, 5‐azacitidine, showed a significant anticancer effect [52]. This suggests that ascorbate's role in potentially augmenting demethylation of DNA could be driving yet another mechanism driving the tantalizing anticancer effects seen in combination with PARP inhibition.

The data presented here contribute to a rapidly growing body of evidence that ascorbate could be an effective weapon in the war against cancer either alone or in combination with other agents; however, there are hurdles yet to clear, namely the lack of high‐quality data from randomized controlled trials. According to clinicaltrials.gov, there are only five actively recruiting trials examining pharmacological ascorbate in cancer, all of which are categorized as Phase 2. The limited data that exist in human subjects are promising, and there is even one study that has looked at PARP inhibition and ascorbate in humans: Eight patients with a variety of stage IV malignancies were treated with either olaparib, niraparib, or talazoparib and pharmacological ascorbate; five patients showed partial response and three showed complete response with no grade three toxicity reported [42]. Results presented herein were used as preclinical data to form the basis for a clinical trial (NCT05501548).

## 5 Conclusions

In summary, this investigation produced molecular and translational evidence that the combination of ascorbate and olaparib could be an effective treatment in mCRPC. Currently, the most evidence‐based mechanism is based on ascorbate generating DNA‐damaging ROS and PARP inhibitors preventing the repair of that damage. However, additional evidence suggests a potentially multifaceted mechanism driving the synergy observed between ascorbate and PARP inhibition based on ascorbate altering cellular energy mechanics and playing a role in epigenetic regulation of the genome. The depth and breadth of these proposed interactions suggest broad applicability and less potential for resistance to occur in response to treatment with ascorbate and PARP inhibition. Currently, there is a dearth of high‐quality data in human subjects, and an emphasis must be placed on performing large‐scale randomized controlled trials as soon as possible. This investigation has produced compelling evidence for the use of ascorbate and PARP inhibition in CRPC, while also contributing to the overall assertion that ascorbate should be considered as an anticancer agent in general.

## Conflict of interest

The contents of this publication are the sole responsibility of the authors and do not necessarily reflect the views, opinions, or policies opinions of the Uniformed Services University of the Health Sciences (USUHS), The Henry M. Jackson Foundation for the Advancement of Military Medicine, Inc. (HJF), the Department of Defense (DoD), or the Departments of the Army, Navy, or Air Force. Mention of trade names, commercial products, or organizations does not imply endorsement by the U.S. Government.

## Author contributions

NG, CJP, WKK, KEK, MJS, and AAS conceived and designed the project. NG, PTG, OIR, NPN, ACM, JJM, ED, IV, and AAS acquired the data. NG, CN, MJS, and AAS analyzed and interpreted the data. NG, MJS, and AAS wrote the paper.

## Supporting information


**Fig. S1.** Manipulating [iron] and [pyruvate] has a significant effect on ascorbate toxicity *in vitro*. (A) Description of the media conditions utilized for the cell lines. (B) C4‐2 (left) or 22Rv1 (right) cells were seeded in DMEM at equal density and allowed to adhere overnight. Sodium pyruvate or iron nitrate was administered, such that the total concentration reached 1 mm for sodium pyruvate or 2 μm for iron nitrate. After 24 h, cells were treated with 1 mm ascorbate; additional sodium pyruvate or iron nitrate was co‐administered in order to maintain the appropriate concentrations. DNA content was quantified using the PicoGreen assay at indicated time points as a means to quantify cell survival. Data are depicted as mean relative cell survival (compared to vehicle control) mean ± SEM of at least three independent biological replicates. (C) C4‐2 cells (left) or 22Rv1 cells (right) were seeded in DMEM on 96‐well plates and allowed to settle overnight. Either sodium pyruvate, iron nitrate or iron chloride was administered to designated wells such that the total concentration reached 1 mm for sodium pyruvate or 2 μm for iron nitrate or iron chloride. After 72 h, DNA content was quantified using the PicoGreen assay. Data are depicted as mean compared to vehicle control ± SEM (standard error of the mean) of at least three independent biological replicates. Statistical significance was determined by Student's *t*‐test. * denotes *P* < 0.05, ** denotes *P* < 0.01, and *** denotes *P* < 0.001.

**Fig. S2.** Pyruvate does not impact olaparib efficacy. (A) C4‐2 cells (IMEM) and 22Rv1 cells (DMEM) were seeded on 96‐well plates and treated with 1 mm ascorbate or 50 μm ascorbate, respectively for 5 days. Cell survival was assessed using the PicoGreen assay. (B) Conditioned C4‐2 cells and 22Rv1 cells were seeded in DMEM on 96‐well plates and pre‐treated with either 1 mm pyruvate or plain DMEM. Cells were treated with 1 μm olaparib (C4‐2 cells) or 5 μm olaparib (22Rv1 cells) for 5 days. Cell proliferation was assessed using the PicoGreen assay. (C) Loewe, HSA, and ZIP synergy scores are depicted for combinational therapy in C4‐2 models. (D–F) C4‐2 cells (IMEM) and 22Rv1 cells (DMEM) were seeded on 96‐well plates and treated with 1 mm ascorbate or 50 μm ascorbate, respectively for 5 days. (D, E) Cells were additionally treated with pyruvate. (E, F) Cells were additionally treated with glutathione (1 mm or 5 mm). Cell viability (D and F) was assessed via Cell Titer Glo and ROS production (E) was assessed. Rescue experiments with pyruvate or glutathione demonstrated suppression of ascorbate‐mediated growth inhibition, supporting ROS‐dependency. (F–H) C4‐2 cells (IMEM) and 22Rv1 cells (DMEM) were seeded on 96‐well plates and treated with 1 mm ascorbate or 50 μm ascorbate, respectively for 5 days. Additionally, cells were treated with (F) PARPi and Pyruvate (1 mm), (G) Paclitaxel, or (H) Doxorubicin, as indicated. Cell survival was assessed using Promega Cell TiterGlow assay. Data are depicted as mean relative cell proliferation (compared to vehicle control) ± SEM of at least three independent biological replicates, with statistical significance was performed using ANOVA with Tukey's *post hoc* test and indicated as **P < 0.01 and ****P < 0.0001.

**Fig. S3.** The *in vivo* combination of olaparib and ascorbate has minimal impact on animal weight. (A, B) C4‐2 xenografts were generated in either non‐castrated (A) or castrated (B) NOD/SCID mice. Mice that developed tumors were randomly assigned into cohorts receiving daily IP injections of normal saline, 50 mg·kg^−1^ olaparib, 4 g·kg^−1^ ascorbate or a combination of olaparib and ascorbate (*n* = 5 mice per group). Tumor volume was measured by calipers and calculated by *V*_tumor_ = (short distance)^2^ × long distance × 0.5236. Mice were weighed once per week to adjust treatment dosing and monitor for toxicity. Data points represent the average of at least three mice per condition. Body weights monitored throughout treatment; no cohort exceeded humane endpoints (>20% loss). Combination treatment consistently delayed tumor growth compared to monotherapies. (C, D) Tumor volume trajectories for intact and castrated mice treated with vehicle, ascorbate, olaparib, or combination. Individual tumor volume plots for the (C) intact and (D) castrated mice with control, single agent, or combination treatment for the course of the experiment. (E) IHC for Ki67, a marker of proliferation, was also assessed to determine whether the observed reduction in tumor volume corresponded to changes in proliferative activity. IHC scoring was performed using the immunohistochemistry (IHC) Profiler package for ImageJ, in which at least three images per tumor were taken and scored for percent Ki67 positivity. Data are depicted as mean ± SD. Statistical comparisons between treatment groups were performed using ANOVA with Tukey's *post hoc* test.

**Table S1.** Detailed media conditions utilized in this study.

## Data Availability

All data generated or analyzed during this study are included in this published article and its supplementary information files. No sequencing datasets were generated for this study.
