# Supplementary figures and images for: PARP inhibition and pharmacological ascorbate demonstrate synergy in castration‐resistant prostate cancer
Source: Mol Oncol. 2026 Jan 14;20(6):1626–42. doi: 10.1002/1878-0261.70183 (PMC13238595; doi:10.1002/1878-0261.70183)

A.

|           | 22Rv1                | C4-2            |
|-----------|----------------------|-----------------|
| Media     | DMEM                 | IMEM            |
| Additives | Ascorbate (50µM-1mM) | Ascorbate (1mM) |

B.

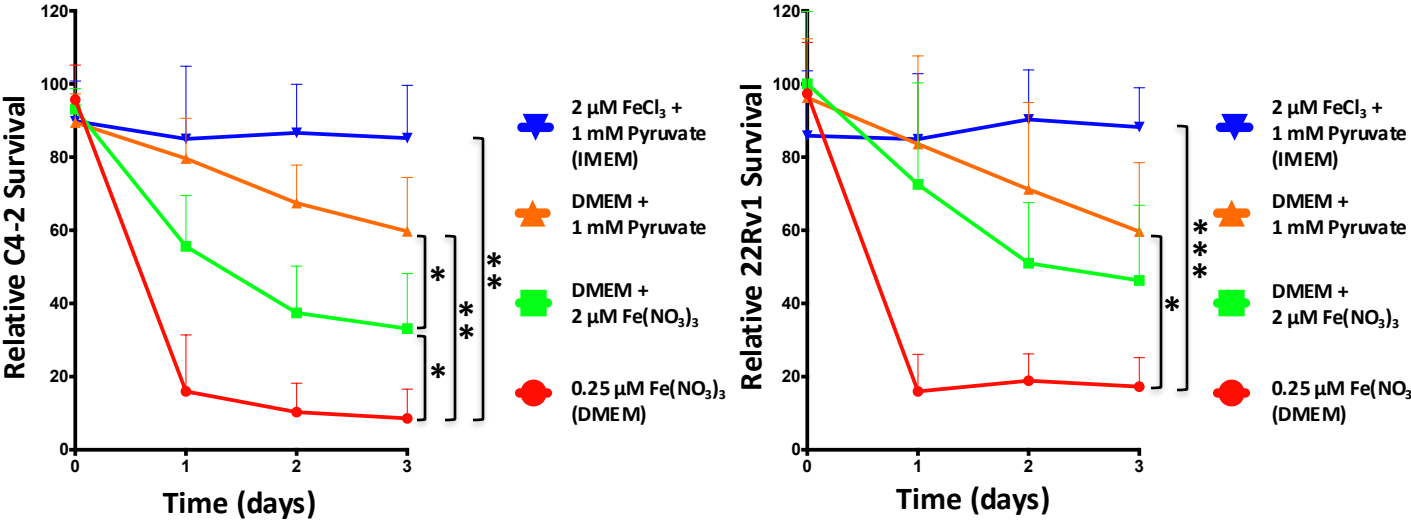

C.

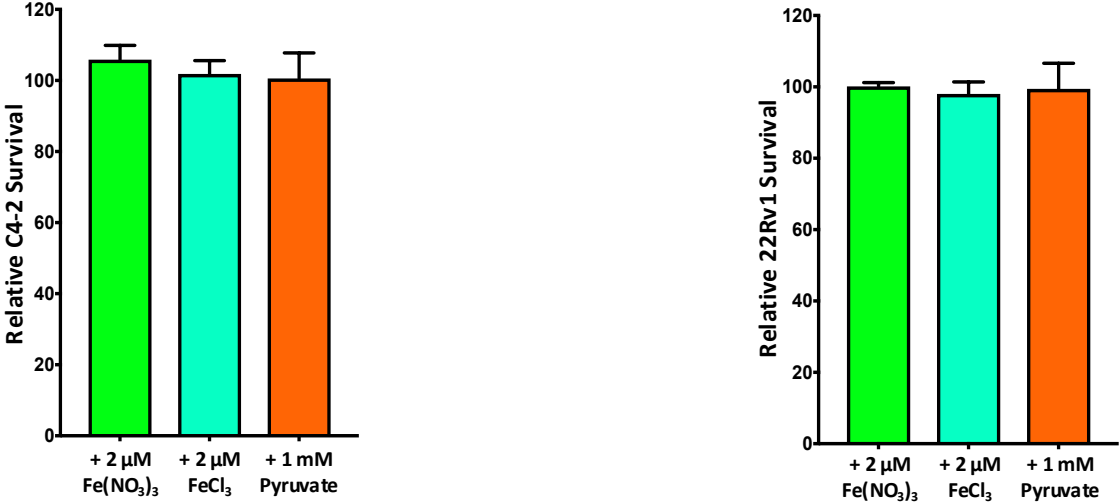

Supplement: Supplementary file 1 — Fig. S1. Manipulating [iron] and [pyruvate] has a significant effect on ascorbate toxicity in vitro. (A) Description of the media conditions utilized for the cell lines. (B) C4‐2 (left) or 22Rv1 (right) cells were seeded in DMEM at equal density and allowed to adhere overnight. Sodium pyruvate or iron nitrate was administered, such that the total concentration reached 1 mm for sodium pyruvate or 2 μm for iron nitrate. After 24 h, cells were treated with 1 mm ascorbate; additional sodium pyruvate or iron nitrate was co‐administered in order to maintain the appropriate concentrations. DNA content was quantified using the PicoGreen assay at indicated time points as a means to quantify cell survival. Data are depicted as mean relative cell survival (compared to vehicle control) mean ± SEM of at least three independent biological replicates. (C) C4‐2 cells (left) or 22Rv1 cells (right) were seeded in DMEM on 96‐well plates and allowed to settle overnight. Either sodium pyruvate, iron nitrate or iron chloride was administered to designated wells such that the total concentration reached 1 mm for sodium pyruvate or 2 μm for iron nitrate or iron chloride. After 72 h, DNA content was quantified using the PicoGreen assay. Data are depicted as mean compared to vehicle control ± SEM (standard error of the mean) of at least three independent biological replicates. Statistical significance was determined by Student's t‐test. * denotes P < 0.05, ** denotes P < 0.01, and *** denotes P < 0.001. [file MOL2-20-1626-s001.pdf]

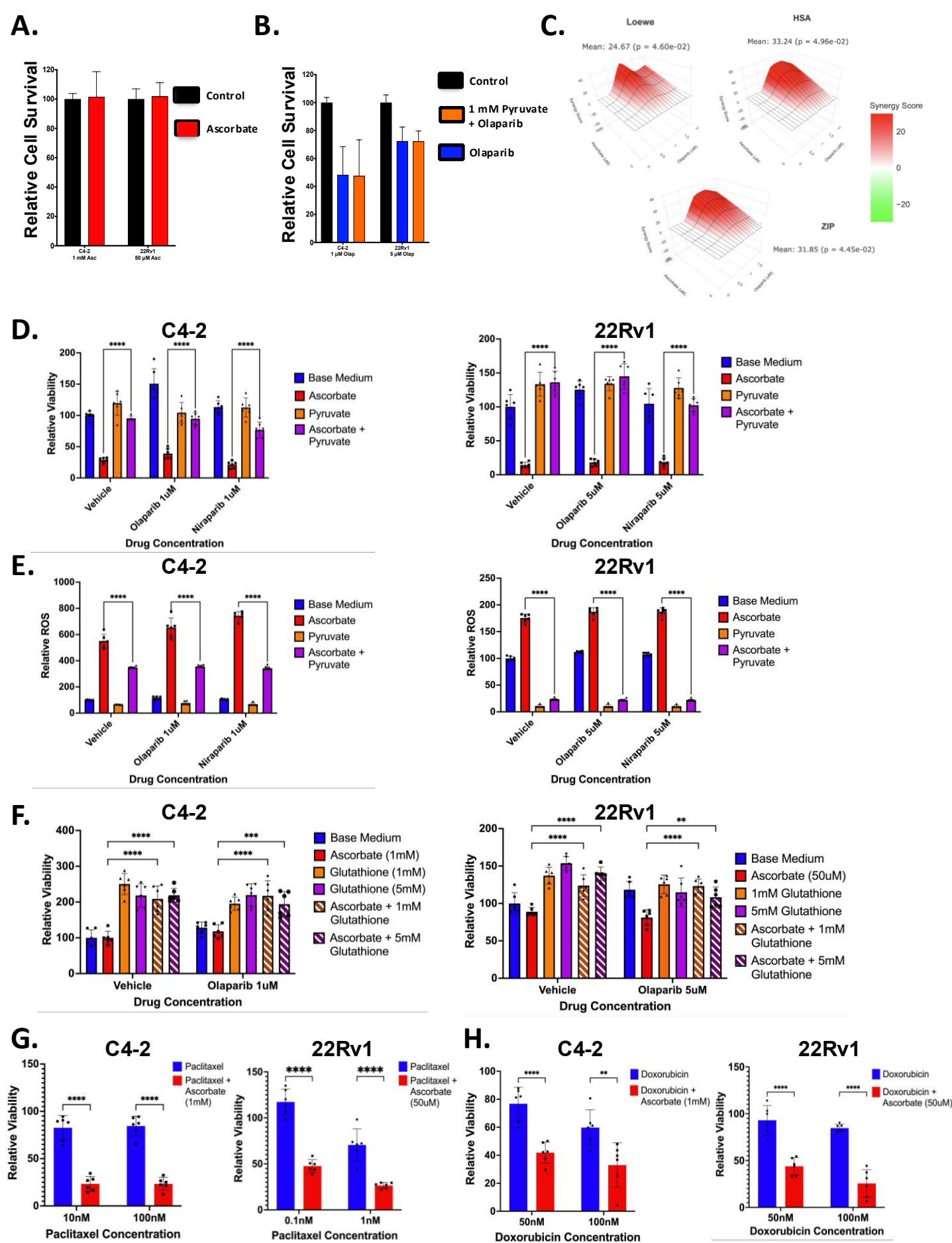

Supplement: Supplementary file 2 — Fig. S2. Pyruvate does not impact olaparib efficacy. (A) C4‐2 cells (IMEM) and 22Rv1 cells (DMEM) were seeded on 96‐well plates and treated with 1 mm ascorbate or 50 μm ascorbate, respectively for 5 days. Cell survival was assessed using the PicoGreen assay. (B) Conditioned C4‐2 cells and 22Rv1 cells were seeded in DMEM on 96‐well plates and pre‐treated with either 1 mm pyruvate or plain DMEM. Cells were treated with 1 μm olaparib (C4‐2 cells) or 5 μm olaparib (22Rv1 cells) for 5 days. Cell proliferation was assessed using the PicoGreen assay. (C) Loewe, HSA, and ZIP synergy scores are depicted for combinational therapy in C4‐2 models. (D–F) C4‐2 cells (IMEM) and 22Rv1 cells (DMEM) were seeded on 96‐well plates and treated with 1 mm ascorbate or 50 μm ascorbate, respectively for 5 days. (D, E) Cells were additionally treated with pyruvate. (E, F) Cells were additionally treated with glutathione (1 mm or 5 mm). Cell viability (D and F) was assessed via Cell Titer Glo and ROS production (E) was assessed. Rescue experiments with pyruvate or glutathione demonstrated suppression of ascorbate‐mediated growth inhibition, supporting ROS‐dependency. (F–H) C4‐2 cells (IMEM) and 22Rv1 cells (DMEM) were seeded on 96‐well plates and treated with 1 mm ascorbate or 50 μm ascorbate, respectively for 5 days. Additionally, cells were treated with (F) PARPi and Pyruvate (1 mm), (G) Paclitaxel, or (H) Doxorubicin, as indicated. Cell survival was assessed using Promega Cell TiterGlow assay. Data are depicted as mean relative cell proliferation (compared to vehicle control) ± SEM of at least three independent biological replicates, with statistical significance was performed using ANOVA with Tukey's post hoc test and indicated as **P < 0.01 and ****P < 0.0001. [file MOL2-20-1626-s002.pdf]

**A.**

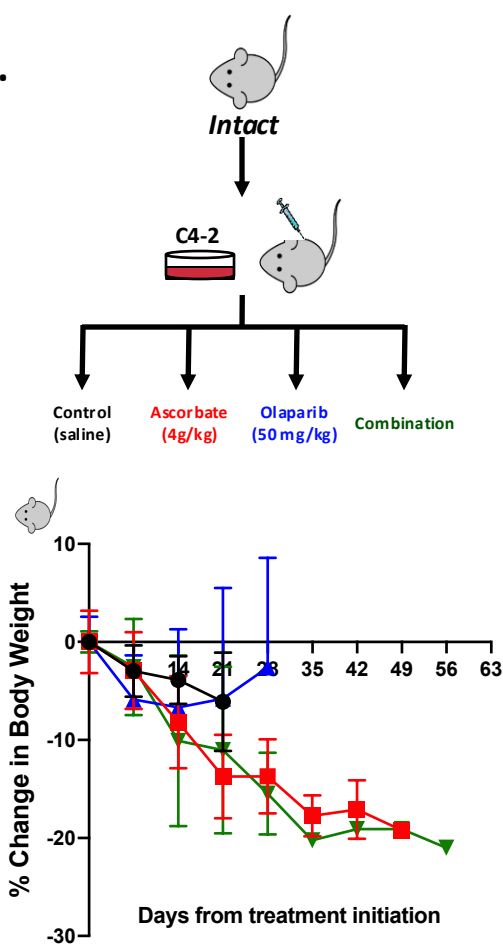

**B.**

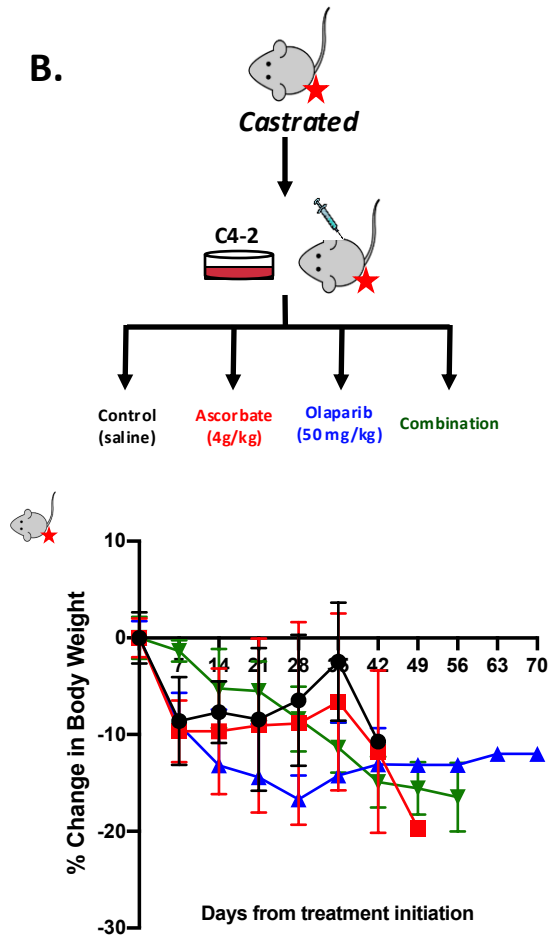

**C.**

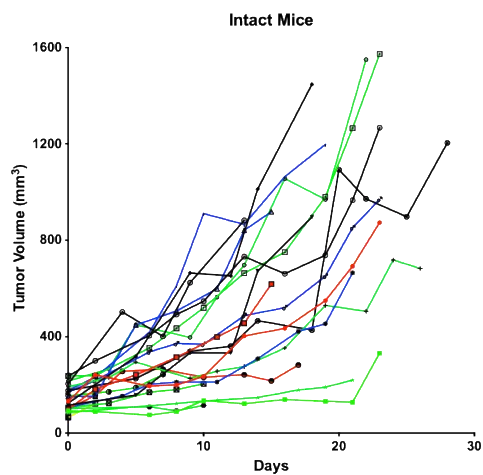

**D.**

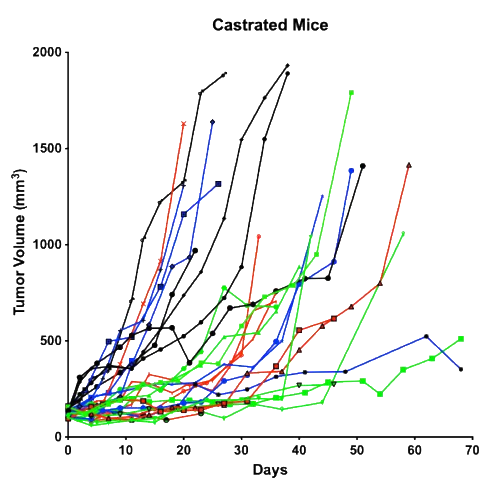

**E.**

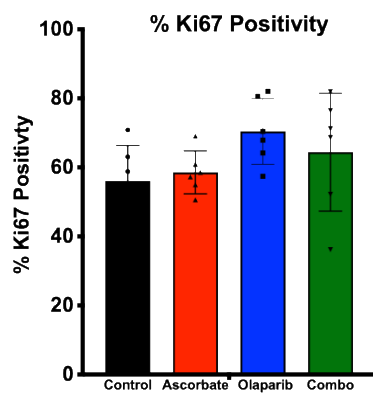

Supplement: Supplementary file 3 — Fig. S3. The in vivo combination of olaparib and ascorbate has minimal impact on animal weight. (A, B) C4‐2 xenografts were generated in either non‐castrated (A) or castrated (B) NOD/SCID mice. Mice that developed tumors were randomly assigned into cohorts receiving daily IP injections of normal saline, 50 mg·kg−1 olaparib, 4 g·kg−1 ascorbate or a combination of olaparib and ascorbate (n = 5 mice per group). Tumor volume was measured by calipers and calculated by V tumor = (short distance)2 × long distance × 0.5236. Mice were weighed once per week to adjust treatment dosing and monitor for toxicity. Data points represent the average of at least three mice per condition. Body weights monitored throughout treatment; no cohort exceeded humane endpoints (>20% loss). Combination treatment consistently delayed tumor growth compared to monotherapies. (C, D) Tumor volume trajectories for intact and castrated mice treated with vehicle, ascorbate, olaparib, or combination. Individual tumor volume plots for the (C) intact and (D) castrated mice with control, single agent, or combination treatment for the course of the experiment. (E) IHC for Ki67, a marker of proliferation, was also assessed to determine whether the observed reduction in tumor volume corresponded to changes in proliferative activity. IHC scoring was performed using the immunohistochemistry (IHC) Profiler package for ImageJ, in which at least three images per tumor were taken and scored for percent Ki67 positivity. Data are depicted as mean ± SD. Statistical comparisons between treatment groups were performed using ANOVA with Tukey's post hoc test. [file MOL2-20-1626-s004.pdf]
