# Supplementary material for: PARP inhibition and pharmacological ascorbate demonstrate synergy in castration‐resistant prostate cancer
Source: Mol Oncol. 2026 Jan 14;20(6):1626–42. doi: 10.1002/1878-0261.70183 (PMC13238595; doi:10.1002/1878-0261.70183)
Supplement: Supplementary file 4 — Table S1. Detailed media conditions utilized in this study. [file MOL2-20-1626-s003.pptx]

## Slide 1
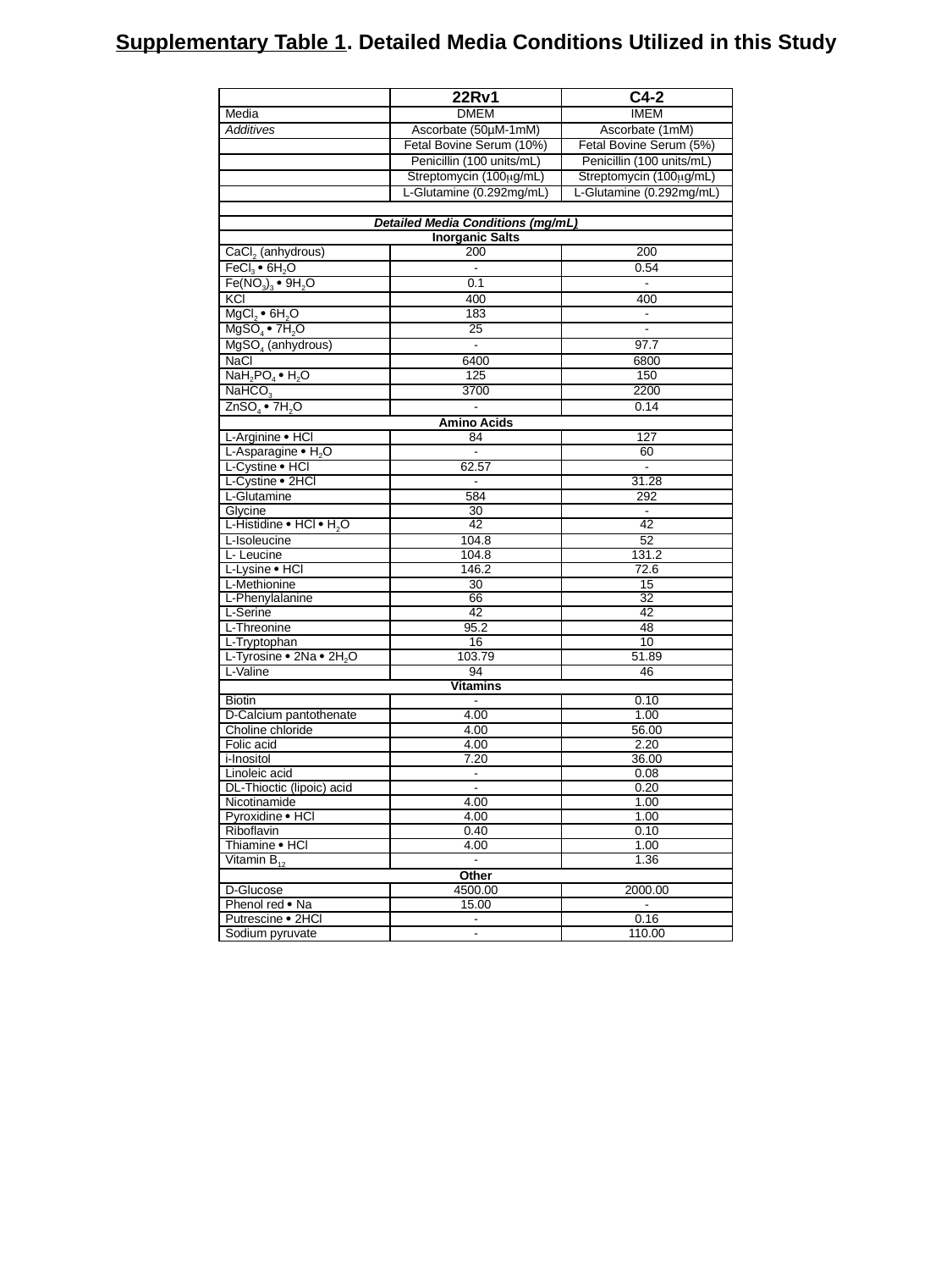

Supplementary Table 1. Detailed Media Conditions Utilized in this Study
| | 22Rv1 | C4-2 |
| --- | --- | --- |
| Media | DMEM | IMEM |
| Additives | Ascorbate (50µM-1mM) | Ascorbate (1mM) |
| | Fetal Bovine Serum (10%) | Fetal Bovine Serum (5%) |
| | Penicillin (100 units/mL) | Penicillin (100 units/mL) |
| | Streptomycin (100μg/mL) | Streptomycin (100μg/mL) |
| | L-Glutamine (0.292mg/mL) | L-Glutamine (0.292mg/mL) |
| | | |
| Detailed Media Conditions (mg/mL) | | |
| Inorganic Salts | | |
| CaCl2 (anhydrous) | 200 | 200 |
| FeCl3  6H2O | - | 0.54 |
| Fe(NO3)3  9H2O | 0.1 | - |
| KCl | 400 | 400 |
| MgCl2  6H2O | 183 | - |
| MgSO4  7H2O | 25 | - |
| MgSO4 (anhydrous) | - | 97.7 |
| NaCl | 6400 | 6800 |
| NaH2PO4  H2O | 125 | 150 |
| NaHCO3 | 3700 | 2200 |
| ZnSO4  7H2O | - | 0.14 |
| Amino Acids | | |
| L-Arginine  HCl | 84 | 127 |
| L-Asparagine  H2O | - | 60 |
| L-Cystine  HCl | 62.57 | - |
| L-Cystine  2HCl | - | 31.28 |
| L-Glutamine | 584 | 292 |
| Glycine | 30 | - |
| L-Histidine  HCl  H2O | 42 | 42 |
| L-Isoleucine | 104.8 | 52 |
| L- Leucine | 104.8 | 131.2 |
| L-Lysine  HCl | 146.2 | 72.6 |
| L-Methionine | 30 | 15 |
| L-Phenylalanine | 66 | 32 |
| L-Serine | 42 | 42 |
| L-Threonine | 95.2 | 48 |
| L-Tryptophan | 16 | 10 |
| L-Tyrosine  2Na  2H2O | 103.79 | 51.89 |
| L-Valine | 94 | 46 |
| Vitamins | | |
| Biotin | - | 0.10 |
| D-Calcium pantothenate | 4.00 | 1.00 |
| Choline chloride | 4.00 | 56.00 |
| Folic acid | 4.00 | 2.20 |
| i-Inositol | 7.20 | 36.00 |
| Linoleic acid | - | 0.08 |
| DL-Thioctic (lipoic) acid | - | 0.20 |
| Nicotinamide | 4.00 | 1.00 |
| Pyroxidine  HCl | 4.00 | 1.00 |
| Riboflavin | 0.40 | 0.10 |
| Thiamine  HCl | 4.00 | 1.00 |
| Vitamin B12 | - | 1.36 |
| Other | | |
| D-Glucose | 4500.00 | 2000.00 |
| Phenol red  Na | 15.00 | - |
| Putrescine  2HCl | - | 0.16 |
| Sodium pyruvate | - | 110.00 |
